# The acute effects of aerobic exercise on sleep in patients with unipolar depression: a randomized controlled trial

**DOI:** 10.1093/sleep/zsab177

**Published:** 2021-07-13

**Authors:** Gavin Brupbacher, Thea Zander-Schellenberg, Doris Straus, Hildburg Porschke, Denis Infanger, Markus Gerber, Roland von Känel, Arno Schmidt-Trucksäss

**Affiliations:** 1 Division of Sports and Exercise Medicine, Department of Sport, Exercise and Health, University of Basel, Basel, Switzerland; 2 OBERWAID AG, St. Gallen, Switzerland; 3 Division of Clinical Psychology and Epidemiology, Department of Psychology, University of Basel, Basel, Switzerland; 4 Division of Sport and Psychosocial Health, Department of Sport, Exercise and Health, University of Basel, Basel, Switzerland; 5 Department of Consultation-Liaison Psychiatry and Psychosomatic Medicine, University Hospital Zurich, Zurich, Switzerland

**Keywords:** depression, mood, exercise, sleep, polysomnography, randomized controlled trial

## Abstract

**Study Objectives:**

Insomnia increases the risk of negative disease trajectory, relapse, and suicide in patients with depression. We aimed at investigating the effects of a single bout of aerobic exercise, performed after 02:00 pm, on the subsequent night’s sleep in patients with depression.

**Methods:**

The study was designed as a two-arm parallel-group, randomized, outcome assessor-blinded, controlled, superiority trial. Patients between 18 and 65 years of age with a primary diagnosis of unipolar depression were included. The intervention was a single 30-minute bout of moderate aerobic exercise. The control group sat and read for 30 minutes. The primary outcome was sleep efficiency measured by polysomnography. Secondary outcomes were other polysomnographic variables, subjective sleep quality, daytime sleepiness, mood states, and adverse events.

**Results:**

Ninety-two patients were randomized to the exercise (*N* = 46) or control group (*N* = 46). There were no clinically relevant differences at baseline. Intent-to-treat analysis ANCOVA of follow-up sleep efficiency, adjusted for baseline levels and minimization factors, did not detect a significant effect of the allocation (*β* = −0.93, *p* = 0.59). There was no evidence for significant differences between both groups in any other objective or subjective sleep outcomes, daytime sleepiness, or adverse events. The intervention had an immediate positive effect on mood states, including depressiveness (*β* = −0.40, *p* = 0.003).

**Conclusions:**

This is the first trial to study the effects of a single bout of aerobic exercise on sleep in patients with depression to the best of our knowledge. Aerobic exercise had no effect on sleep efficiency but had a strong beneficial effect on mood and did not increase adverse outcomes. These results add to the growing body of evidence that, contrary to sleep hygiene recommendations, exercise after 02:00 pm is not detrimental for sleep.

**Clinical Trial Registration:**

Clinicaltrials.gov, https://clinicaltrials.gov/ct2/show/NCT03673397. Protocol registered on September 17, 2018.

**Statement of Significance**

Insomnia is a core symptom of unipolar depression. This is the first trial to study the acute effects of aerobic exercise on sleep in patients with depression to the best of our knowledge. We found no evidence for an effect of allocation on sleep-related variables or adverse effects. However, a single session of moderate aerobic exercise substantially improved mood states.

## Introduction

Insomnia is a core symptom of unipolar depression, which critically predicts depression onset, trajectory, and recurrence. Insomnia is defined as having difficulties initiating or maintaining sleep, early morning awakening and is often accompanied by daytime impairments [1]. Meta-analysis has shown that insomnia more than doubles the risk for depression [2]. This risk might be mainly driven by difficulties initiating sleep, as was suggested by a recent network outcome analysis [3]. Epidemiological studies have found insomnia prevalence rates of up to 90% in patients with depression [4]. Longitudinal studies have repeatedly shown a bidirectional link between insomnia and depression [5]. Insomnia symptoms negatively affect the disease trajectory [6]. It reduces the responsiveness to psychotherapy [7], pharmacotherapy, or a combination of these [8, 9]. It also seems to increase the risk of developing treatment-resistant depression [10], suicidal behavior [11], as well as myocardial infarction [12]. Sleep complaints are one of the most common symptoms after remission [13]. Residual insomnia is problematic because insomnia is a prodromal symptom [14], thereby increasing the risk for depression relapse [15]. There is a need to develop additional treatments for insomnia in patients with depression, considering the evidence presented above.

Moderate aerobic exercise might be a viable candidate as an adjuvant therapy for insomnia in patients with depression. Aerobic exercise is a rhythmic activity that involves large muscle groups and that primarily uses aerobic energy-producing systems. Moderate aerobic exercise refers to intensities of 55%–69% maximal heart rate, 40%–59% heart rate reserve, or 11–13 rate of perceived exertion [16]. Regular moderate aerobic exercise has positive effects on subjective sleep quality in patients with depression, as we have demonstrated in a recent network meta-analysis [17]. These effects are particularly strong when aerobic exercise is combined with treatment as usual. Regular moderate aerobic exercise also improves symptoms of depression [18]. Furthermore, chronic aerobic exercise improves cardiorespiratory fitness in patients with depression [19]. The effect on fitness is pertinent because depression increases the risk of coronary heart disease and myocardial infarction [20]. Current general sleep hygiene guidelines recommend regular exercise before 02:00 pm [21]. The authors of this guideline argue that vigorous exercise before bedtime causes the release of endorphins which can delay the onset of sleep [21]. This caveat on timing severely limits the feasibility of exercise interventions. Moreover, epidemiological data [22] and meta-analysis of randomized controlled trials in healthy individuals [23] have shown that there is no adverse effect of an exercise bout after 02:00 pm on sleep. This reflects the lack of evidence concerning the *acute* effects of moderate exercise on sleep, especially in patients with depression.

The primary goal of this trial was, therefore, to investigate the effects of a single bout of moderate aerobic exercise on the subsequent night’s sleep efficiency in patients with depression. For the duration of the exercise bout, we chose 30 min, corresponding to the recommendation of the American College of Sports Medicine and the American Heart Association for daily physical activity with beneficial health effects [24]. Secondary goals were to investigate the intervention’s effect on other polysomnographic outcomes, adverse outcomes, daytime sleepiness, and mood. We hypothesized that the intervention improves (1) sleep efficiency, (2) sleep continuity, (3) sleep architecture, (4) subjective sleep quality, (5) daytime sleepiness, and (6) mood. We expected no evidence for an effect on the frequency and the severity of adverse events as an exploratory outcome.

## Methods

### Trial design

This was a two-arm parallel-group, randomized, outcome assessor-blinded, controlled, superiority trial. The trial took place in the psychosomatic in-patient rehabilitation unit of the OBERWAID AG, a rehabilitation clinic in St. Gallen, Switzerland. The Ethics Committee East Switzerland, St. Gallen, Switzerland, approved the study protocol (EKOS 18/089). We prospectively registered this trial in the clinicaltrial.gov registry on September 17, 2018 (NCT03673397). A detailed study protocol that clearly states the study’s rationale is available [25]. There were no amendments and no deviations from the protocol. This report follows the CONSORT guideline for randomized controlled trials [26]. The data underlying this article is available in the Harvard Dataverse at https://doi.org/10.7910/DVN/WASN36 and will be shared at reasonable request to the corresponding author.

## Procedure and Assessments

### Screening

**Figure 1.**
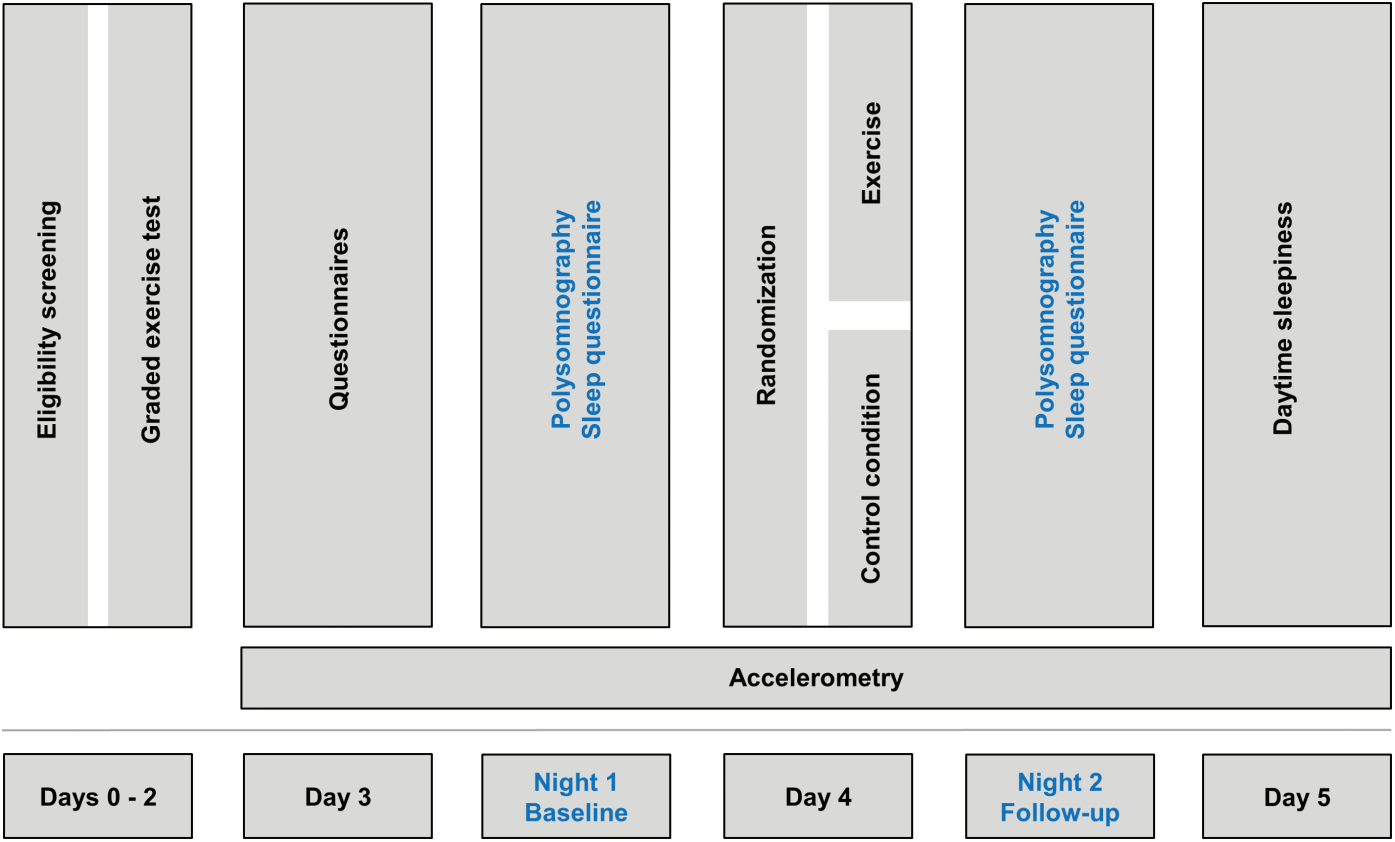
Trial design.

Patients admitted to the in-patient psychosomatic rehabilitation unit of the OBERWAID clinic were screened for inclusion. The trial took place in the first 5 days of the patient’s psychosomatic in-patient rehabilitation, see Figure 1. The first author or another representative of the OBERWAID AG obtained written informed consent from participants. Eligibility criteria are listed in Table 1. We provide a detailed rationale for the inclusion and exclusion criteria in the study protocol [25].

**Table 1.** Inclusion and exclusion criteria

**Inclusion criteria**
• ≥18 and ≤65 years old
• Primary diagnosis of depression (F32, F33) without psychotic episode according to ICD-10
**Exclusion criteria**
• Regular use of hypnotic agents* (patients are included if no hypnotic agents were taken 2 weeks before study participation)
• Factors precluding exercise testing or training†
• Use of beta-blockers (except Carvedilol & Nebivolol)
• Use of opioids
• History of epilepsy
• Restless legs syndrome defined by ≥7 points on the Restless Legs Screening Questionnaire [35]
• Moderate or severe sleep apnea defined by an oxygen desaturation index (using 4% criterion) ≥ 15 in the baseline polysomnography
• Morbid adiposity with BMI > 40

*Hypnotic agents are defined as follows: orexin receptor agonists, benzodiazepine receptor agonists, sedating antidepressants, neuroleptics, benzodiazepines, melatonin agonists, heterocyclics, anticonvulsants, over the counter sleep aids (sedating antihistamines, melatonin L-tryptophan, valerian), and cannabinoids.

^†^Absolute and relative contraindications are based on ACSM’s Guidelines for Exercise Testing and Prescription.

ICD-10, International classification of diseases, version 10; BMI, body mass index.

After providing informed consent, we formally screened patients. The screening included a consultation with an experienced psychiatrist and a full history and medical examination by an experienced internist. Patients with undiagnosed sleep apnea were excluded according to the baseline polysomnography.

### Graded exercise testing

Patients fulfilling all eligibility criteria assessed thus far (sleep apnea criterion is determined later by polysomnography, see Figure 1) performed sub-maximal graded exercise testing on a bicycle ergometer (ergoselect 200, Ergoline, Bitz, Germany). We determined the anaerobic threshold according to the method of Dickhuth et al. [27] using a specialized software program (Ergonizer, Freiburg, Germany). A detailed description of the graded exercise testing can be found in the study protocol [25].

### Patient characterization

We administered multiple questionnaires to characterize patients at baseline. We assessed somatic multimorbidity with the Patient Health Questionnaire Somatic Symptom Scale (PHQ-15) [28] and the Modified Cumulative Illness Rating Scale (CIRS) [29]. The PHQ-15 is a self-administered questionnaire with 15-items. It measures the severity of somatic symptoms (e.g. back pain) within the previous 4 weeks on a three-point Likert scale (*0 = not bothered at all to 2 = bothered a lot*). The symptoms in this questionnaire account for more than 90% of physical complaints reported in outpatient settings and its validity has been demonstrated [28]. The CIRS provides physician-rated scores of multimorbidity. It measures the severity of symptoms over 14 organ systems (e.g. heart) on a five-point scale (*0 = no problem* to *4 = extremely severe problem*). Inter-rater agreement and validity have been demonstrated [29]. We measured depressive symptom severity with the Patient Health Questionnaire-9 (PHQ-9) [30]. The nine symptom items (e.g. “Feeling down, depressed, or hopeless”) are scored on a four-point Likert scale (*0 = not at all to 3 = nearly every day*). The psychometric properties, including the validity of the cutoffs from mild to severe depression, have been demonstrated [30]. Anxiety was assessed using the Hospital Anxiety and Depression Scale (HADS) [31]. This questionnaire measures anxiety with seven items (e.g. *“I get sudden feelings of panic”*), each on a four-point Likert scale (e.g. *0 = not at all to 3 = most of the time*). Psychometric properties, including diagnostic test accuracy, have been demonstrated [31, 32]. Stress was assessed with the Perceived Stress Scale [33]. This questionnaire operationalizes stress as the degree to which life is experienced as unpredictable, uncontrollable, and overloaded in the past month. The ten items (e.g. *“In the last month, how often have you felt nervous and stressed?”*) are scored on a five-point Likert scale (*0 = never* to *4 = very often*). The psychometric properties have been demonstrated [33]. Sleep reactivity was assessed with the Ford Insomnia Response to Stress Test [34, 35]. The nine items of this self-report questionnaire assess the likelihood of sleep disturbances in response to stressful situations (e.g. “*How likely is it for you to have difficulty sleeping after an argument”*) on a four-point Likert scale (*1 = not very likely* to *4 = very likely*). Reliability and validity have been demonstrated [36]. Dysfunctional sleep-related thoughts and attitudes were assessed with the Short form of the Dysfunctional Beliefs and Attitudes about Sleep Scale (DBAS) [37]. The sixteen items (e.g. *“I am worried that I may lose control over my ability to sleep.”*) are rated on a Likert scale (*0 = strongly disagree* to *10 = strongly agree*). The reliability and validity of this questionnaire have been demonstrated [37]. Chronotype was assessed using the Morningness-Eveningness Questionnaire [38]. The 19 multiple-choice questions (four- or five-point scale) assess sleep habits and propensity for performance throughout the day. The sum score (range: 16 to 86) can be translated into chronotype (<42, evening type; 42–58, neither; >58, morning type). Adequate psychometric properties have been demonstrated [39, 40]. We measured chronic daytime sleepiness using the Epworth Sleepiness Scale [41]. The likelihood of dozing off in eight daily situations (e.g. watching television) is assessed on a four-point Likert scale (*0 = would never doze* to *3 = high chance of dozing*). The reliability and validity have been demonstrated [42]. Subjective sleep disturbance was measured with the Pittsburgh Sleep Quality Index [43, 44]. This 18 item scale assesses subjective sleep quality, sleep latency, sleep duration, habitual sleep efficiency, sleep disturbances, use of sleeping medication, and daytime dysfunction. The sum score with a cutoff value of ≥5 has been shown to distinguish good and poor sleepers [45].

Since patients cannot be blinded in exercise trials and considering the importance of patient preference and satisfaction [46, 47], we also assessed credibility and expectancy on day three (i.e. before randomization) using two items (adapted from [48, 49]): “At this point, how logical does the therapy offered to you seem?”, “At this point, how successfully do you think this treatment will be in reducing your insomnia symptoms?.” Patients rated the credibility and expectancy items on a four-point Likert scale (*1* = *not at all* to *4 = very*).

### Baseline assessments

We assessed multiple objective and subjective sleep outcomes at baseline. We performed the baseline polysomnography on the night before the intervention to exclude patients with at least moderate sleep apnea (oxygen desaturation index ≥ 15) and assess potential first-night effects. We recorded polysomnographic data with the SOMNOscreen™ plus RC (Somnomedics, Randersacker, Germany) using the following montage: one EEG channel (Fp2-A1, 512 Hz), two EOG channels (512 Hz), one EMG channel, (512 Hz), one ECG channel (modified lead II, 512 Hz), thoracic respiratory effort channel (inductance plethysmography belt, 32 Hz), finger photoplethysmography (nondominant arm, 128 Hz), body position (stored every 30 s), movement (32 Hz), and ambient light (stored every 30 s). The validity of this montage for the assessment of sleep stages has previously been demonstrated [50]. Polysomnography recordings were scored independently by two trained scorers according to the American Association of Sleep Medicine guidelines [51]. All polysomnographic variables were calculated according to the American Association of Sleep Medicine guidelines [51]. Both scorers have demonstrated good agreement with the gold standard ratings in the AASM inter-scorer program [52]. Their average agreement with the gold standard was 88% and 84%, respectively, which is above average [53]. Scorers were blinded against allocation, time points, and each other’s ratings. The subjective sleep quality of the baseline night was measured upon awakening with the revised *Schlaffragebogen A* [54], a German sleep questionnaire recommended by guidelines [55]. This self-report questionnaire contains 25 items that load onto five factors: sleep quality, recuperation after sleep, calmness before sleep, exhaustion before sleep, and nocturnal psychosomatic symptoms. Internal consistency, factor structure, and validity have been demonstrated [54].

### Randomization

Once eligibility was confirmed by baseline polysomnography, patients were randomly allocated to one of both groups using minimization (see Figure 1). We used a nondeterministic unweighted minimization algorithm [56] with a random element of 0.8. The allocation ratio was 1:1. We used minimization to increase the probability of balanced groups across the following predictive factors: sex, age, depression severity (PHQ-9 score), and subjective sleep quality (PSQI score). Allocation concealment consisted of four aspects: (1) requesting randomization after baseline measurement, (2) using a random element, (3) requesting allocation for participants by four different study nurses, and (4) not disclosing full details of minimization to study nurses in accordance with the SPIRIT guideline [57]. Further details, including the rationale for the selection of minimization factors, are provided in Section 1 of the Supplementary Material.

### Intervention and control condition

The exercise and control interventions started at approximately 04:45 pm in the afternoon. Patients allocated to the intervention group performed a single bout of supervised aerobic exercise on a bicycle ergometer (ergoselect 200, Ergoline, Bitz, Germany). The intervention began with a 5-minute warm-up period in which the intensity was gradually increased. Thereafter, patients exercised at an intensity of 80% of the individual anaerobic threshold (i.e. as defined by graded exercise testing) for 30 min (i.e. as recommended by guidelines [24]). We recorded average Watt and heart rate (Polar H7 chest strap, Polar OY, Finland) as well as perceived exertion in the 5th, 15th, and 30th min in the exercise group. Patients allocated to the control group were asked to sit and read magazines when the intervention group was exercising. All patients completed a mood questionnaire (*Befindlichkeitsskala*) [58] directly before and after the intervention. This questionnaire consists of 40 adjectives (e.g. cheerful, sad) with a five-point Likert scale (*1 = not at all* to *5 = very much*) to indicate the experience of these adjectives in the present moment. Items load onto eight subscales (with five items each): activity, elation, contemplation, calmness, fatigue, depression, anger, and excitement. We also administered six Likert scaled (*1 = not at all* to *5 = very much*) questions on adverse outcomes (pain, dizziness, cardiovascular symptoms, respiratory symptoms, nausea, and “other”) immediately after the intervention.

We took multiple measures to offset the risk of performance bias that is inherent to exercise trials. Patients were instructed not to perform any other physical exercise except their daily activities. All patients wore an accelerometer (vivofit 2, Garmin, Schaffhausen, Switzerland) on their nondominant wrist on the days before and after the sleep assessments. The validity of this is accelerometer has been demonstrated [59]. The accelerometer data allowed us to gauge potential contamination through other physical activity. Moreover, the rules and schedules of the in-patient rehabilitation clinic (e.g. timing of meals, consumption of multimedia, and alcohol) limit the variability of many behavioral aspects and ancillary treatments which could influence sleep.

### Follow-up assessments

We repeated objective and subjective sleep assessments at follow-up identically to baseline (see Figure 1). Also, we administered the adverse outcomes question upon awakening. Lastly, the Stanford Sleepiness Scale [60] was administered four times (08:00 am, 12:00 pm, 04:00 pm, 08:00 pm) on the day after the intervention to assess excessive daytime sleepiness.

### Outcomes

Polysomnographic sleep efficiency was the primary outcome. We chose a polysomnographic variable because the inability to blind patients against allocation in exercise trials increases the risk of a detection bias for patient-reported outcomes. Patients with depression have difficulties initiating and maintaining sleep, and they also show early morning awakening [61]. Sleep efficiency is an appropriate polysomnographic measure to capture these sleep problems. We defined multiple secondary outcomes which should help to inform clinical decision-making. Secondary polysomnographic outcomes were: total sleep time (TST), sleep onset latency (SOL), wake after sleep onset (WASO), number of awakenings (NA), stage one sleep (N1; in percent of TST), stage two sleep (N2; in percent of TST), stage three sleep (N3; in percent of TST), non-REM sleep (in percent of TST), REM sleep (in percent of TST), REM-sleep latency (minutes), and stage shift index (stage changes per hour). Secondary subjective outcomes were subjective sleep quality, daytime sleepiness, mood states, and adverse events. The secondary outcomes of presleep arousal and nocturnal autonomic cardiovascular modulation prespecified in the protocol will be published in a different paper.

### Statistical methods

We analyzed the primary outcome using an ANCOVA model. Thereby, we used baseline sleep efficiency and minimization factors [62] as covariates, allocation as the independent variable, and follow-up sleep efficiency as the dependent variable. First, we checked the statistical prerequisites. If residuals were heteroscedastic, we used heteroscedasticity-consistent estimation of the covariance matrix (HC3) [63]. We used intent-to-treat analysis to reduce attrition bias. We replaced missing values using multiple imputations [64]. Sensitivity analyses for the primary outcome were performed to gauge the influence of several factors: influential data points, per-protocol analysis (to reflect optimal adherence to treatment), missing data (analysis of complete data only), and chronotypes. All analyses were performed using the software R, version 3.6.3 [65].

Sample size calculation was performed according to the procedure defined by Borm et al. [66]. The expected treatment effect is based on the work of Passos et al. [67], which found a standardized mean difference in polysomnographic sleep efficiency of 0.53 (detailed rationale for the choice of this effect size is provided in the protocol [25]). With a power of 0.8 and a two-sided alpha of 0.05, 57 subjects would be required for each group using a *t*-test. According to the method of Borm et al., this sample size can be multiplied by a “design factor” of (1 − *ρ*^2^), where *ρ* is the correlation coefficient between baseline and follow-up outcome [66]. We used a conservative estimate and let *ρ* = 0.5, resulting in a “design factor” of 0.75 (1 − 0.5^2^ = 0.75). Hence the sample size needed per group is 43 (57 × 0.75 = 42.75). We anticipated a dropout rate of approximately 7% (half the average dropout rate of trials investigating the chronic effects of exercise in patients with depression [68]). Therefore, we calculated the total sample size to be 92 (2 × 43 × 1.07 = 92).

The choice of statistical analysis for secondary outcomes was based on the number of assessments for each outcome. We calculated the effect of allocation using ANCOVA models (as outlined for the primary outcome) for all outcomes assessed at baseline and follow-up. We analyzed acute daytime sleepiness (four measurements) using a two-way repeated-measures ANOVA with Benjamini-Hochberg [69] corrected post hoc paired sample *t*-tests. We assessed adverse outcomes using Mann-Whitney-U tests. The threshold for statistical significance was set at *p* ≤ 0.05. We did not adjust secondary analyses for multiple testing, and thus these should be considered exploratory.

## Results

**Figure 2.**
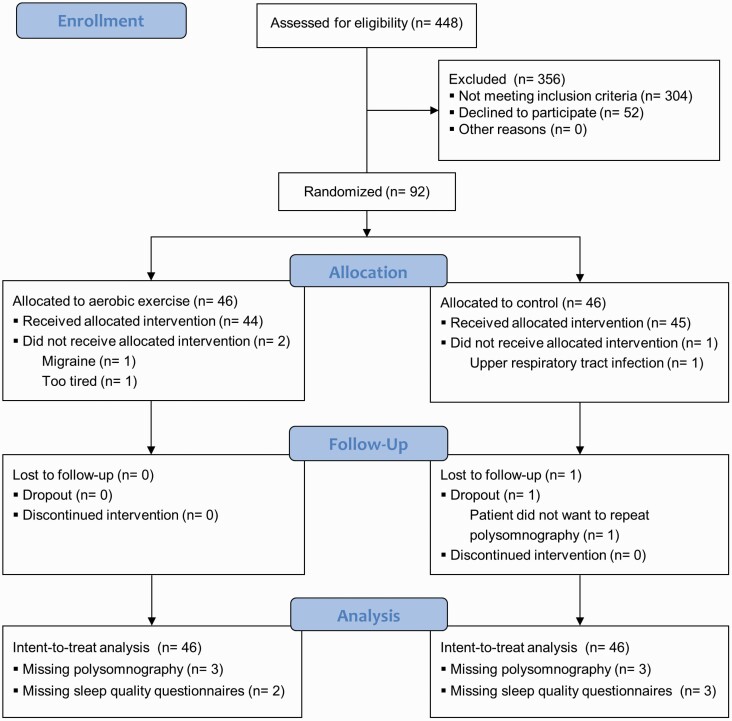
CONSORT participant flow.

Four hundred and forty-eight patients were screened for inclusion between September 2018 and January 2020 (see Figure 2). The most frequent reason for exclusion was the use of hypnotics (48%), followed by exercise contraindications (13%), and not being diagnosed with unipolar depression (8%). Ninety-two patients met eligibility criteria and were allocated to moderate aerobic exercise (*N* = 46) or the control condition (*N* = 46). Baseline characteristics of the study sample are summarized in Table 2. Demographic and clinical characteristics were well balanced at baseline. Four patients did not complete the study (two in each group). Dropouts did not seem to differ from completers at baseline. In addition to the dropouts, two polysomnographic measurements at follow-up failed (one in each group), and five patients did not complete the subjective sleep questionnaires. Inter-rater reliability was good (Cohen’s Kappa: wake: 0.82; N1: 0.49; N2: 0.68; N3: 0.73; REM: 0.79). The intervention was implemented as planned: the mean rate of perceived exertion was 13.6 (*SD* = 1.6), and the mean percent of age-predicted maximal heart rate over the course of the intervention was 70.6% (*SD* = 6.8%), see Supplementary Figures S1 and S2. There was no evidence to suggest that average daily steps differed between the groups, *F*(1.82, 144.14) = 0.08, *p* = 0.9.

**Table 2.** Patient characteristics at baseline

		Control (*N* = 46)	Exercise (*N* = 46)
Age		47.50 [43, 51]	46.00 [37, 53]
Sex	Female	33 (71.7)	32 (69.6)
	Male	13 (28.3)	14 (30.4)
BMI		24.4 [22.2, 27.7]	25.0 [21.8, 29.2]
PHQ15		12.5 [8.0, 14.0]	12.5 [8.3, 15.8]
CIRS		3.0 [1.3, 5.0]	3.5 [2.0, 4.8]
PHQ9		15.0 [12.0, 17.0]	14.0 [12.0, 17.0]
HADS anxiety		11.5 [9.0, 14.0]	11.0 [9.3, 14.0]
PSS10		26.0 [21.3, 28.0]	27.0 [22.0, 29.0]
MEQ		56.5 [47.0, 61.8]	51.0 [45.3, 59.0]
DBAS		4.8 [3.8, 5.4]	4.6 [3.6, 5.7]
FIRST		27.5 [21.3, 29.8]	27.0 [24.0, 29.8]
ESS		10.0 [7.0, 12.0]	9.0 [6.0, 11.0]
PSQI		9.5 [6.3, 12.0]	10.0 [7.00, 13.75]
Oxygen desaturation index*		1.8 [0.7, 3.9]	2.1 [0.7, 3.9]
Sleep efficiency*		88.8 [82.5, 94.0]	91.3 [84.4, 93.5]
Total sleep time*		416.7 [382.3, 463.1]	439.0 [393.3, 479.5]
Sleep onset latency*		14.5 [6.8, 27.2]	14.0 [5.5, 23.3]
Wake after sleep onset*		37.8 [19.1, 62.6]	30.8 [18.0, 43.3]
Number of awakenings*		15.8 [11.4, 20.8]	17.0 [13.5, 23.5]

*Measured polysomnographically.

Continuous variables are presented as medians with interquartile ranges (median [Q_1_, Q_3_]) and sex is presented as absolute numbers and percentages (*N* (%)).

BMI, body mass index; PHQ15, Patient Health Questionnaire 15; CIRS, Cumulative Illness Rating Scale; PHQ9, Patient Health Questionnaire 9; HADS anxiety: Hospital Anxiety and Depression, anxiety subscale; PSS10, Perceived Stress Scale, 10 item version; MEQ, Morningness-Eveningness Questionnaire; DBAS, Dysfunctional Beliefs and Attitudes about Sleep Scale; FIRST, Ford Insomnia Response to Stress Test; ESS, Epworth Sleepiness Scale; PSQI, Pittsburgh Sleep Quality Index.

**Figure 3.**
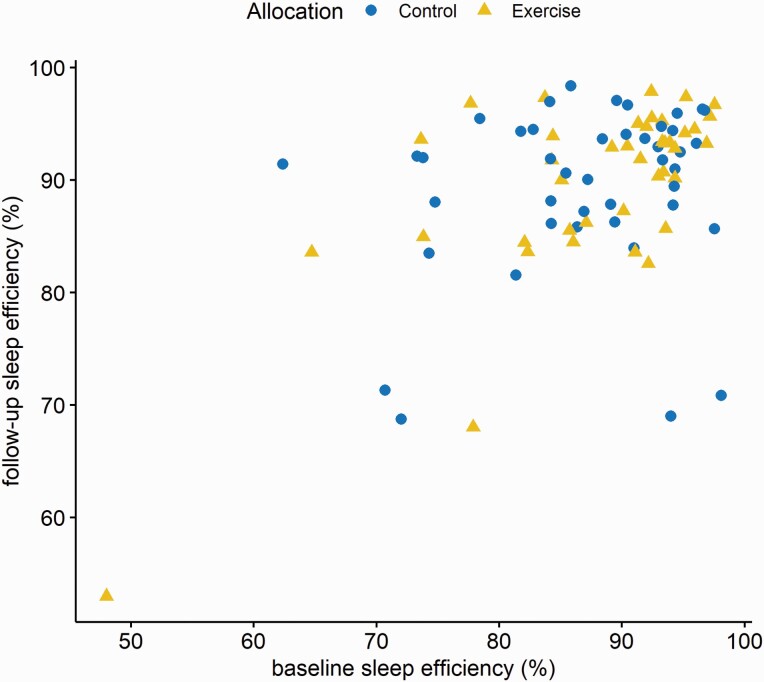
Baseline and follow-up sleep efficiency by allocation.

Intent-to-treat analysis ANCOVA of follow-up sleep efficiency, adjusted for pre-intervention levels and minimization factors did not detect a significant effect of the allocation, see Table 3 and Figure 3. The coefficient for allocation is the difference between the mean change scores of each group.

**Table 3.** ANCOVA table for intent-to-treat analysis of sleep efficiency at follow-up

Term	Estimate (*β*)	Standard error (*β*)	95% confidence interval		*P*
Intercept	59.50	18.79	22.09	96.91	0.002
Baseline sleep efficiency	0.42	0.19	0.03	0.80	0.03
Age	−0.05	0.10	−0.25	0.15	0.61
Sex (male*)	−0.98	1.87	−4.70	2.74	0.60
PHQ9 score	−0.20	0.22	−0.64	0.23	0.36
PSQI score	−0.08	0.22	−0.52	0.37	0.73
Allocation (exercise†)	−0.93	1.70	−4.32	2.47	0.59

*Sex was coded as follows: 1 = male, 2 = female.

^†^Exercise was coded as follows: 1 = control, 2 = exercise.

Age, sex, PHQ9 score, and PSQI score were entered as covariates because they were used as minimization factors.

PHQ9, Patient Health Questionnaire 9; PSQI, Pittsburgh Sleep Quality Index.

This finding was robust in all sensitivity analyses. Prespecified sensitivity analyses included per protocol (i.e. only including patients who reported an RPE (rate of perceived exertion) value of ≥13 at the end of the exercise intervention) and complete case (i.e. not using imputed data) analyses. Furthermore, we identified one influential data point (based on Cook’s distance and DFBETA), clearly visible in Figure 3. Excluding this observation did not alter the primary ANCOVA results or any of the other aforementioned sensitivity analyses. There were no significant interaction effects of chronotype (*β* = 0.20, 95% CI = −0.08 to 0.47, *p* = 0.19), expectancy (*β* = −2.33, 95% CI = −8.43 to 3.79, *p* = 0.45), and credibility (*β* = −4.17, 95% CI = −12.54 to 4.19, *p* = 0.32) with allocation. There was no evidence that the rate of perceived exertion (*r*_s_ = 0.05, *p* = 0.76) nor the average percent of age-predicted maximal heart rate (*r*_s_ = −0.16, *p* = 0.30) was associated with sleep efficiency in the intervention group at follow-up. Steps on day four (measured by accelerometer) did not predict sleep efficiency at follow-up in the ANCOVA model (*β* = −0.10, 95% CI: −0.46 to 0.25, *p* = 0.56; for ease of interpretation, step count was divided by 1,000).

There was no evidence for an effect of allocation on any other objectively or subjectively measured sleep outcomes. The effect of allocation on polysomnographic and subjective sleep outcomes are summarized in Tables 4 and 5, respectively. The internal consistency of the sleep questionnaire subscales was adequate (Cronbach’s alpha: sleep quality = 0.87; recuperation after sleep = 0.93; mental balance before sleep = 0.90; exhaustion before sleep = 0.68; nocturnal psychosomatic symptoms = 0.65). There was no evidence for an effect of allocation on daytime sleepiness over time, *F*(3, 222) = 1.15, *p* = 0.33. Post-hoc tests assessing the effects of allocation were not significant (see Supplementary Figure S3).

**Table 4.** Coefficients of exercise allocation in ANCOVA models predicting polysomnographic outcomes

Outcome	Estimate for exercise allocation* (*β*)	Standard error (*β*)	95% Confidence interval		*P*
Total sleep time	−0.50	11.86	−24.09	23.10	0.97
Sleep onset latency	−1.15	3.39	−7.89	5.60	0.74
Wake after sleep onset	1.86		−11.32	15.04	0.78
Number of awakenings	−1.70	1.28	−4.24	0.84	0.19
Stage shift index	0.04	0.61	−1.18	1.26	0.95
N1 (% TST)	−1.4	0.91	−3.21	0.42	0.13
N2 (% TST)	0.94	1.64	−2.34	4.21	0.57
N3 (% TST)	0.55	1.32	−2.07	3.16	0.68
REM (% TST)	−0.02	1.10	−2.21	2.18	0.99
REM latency	4.20	10.76	−17.23	25.64	0.70

*Allocation was coded as follows: 1 = control, 2 = exercise.

All models used baseline values of the outcome as well as minimization factors (sex, age, PHQ9 score, and PSQI score) as covariates and allocation as the independent variable. The coefficient for allocation is the difference of the mean change score in the exercise group compared to the control group.

N1, stage one sleep, N2, stage two sleep, N3, stage three sleep, REM, rapid eye movement sleep, TST, total sleep time.

**Table 5.** Coefficients of exercise allocation in ANCOVA models predicting subjective sleep outcomes

Outcome	Estimate for exercise allocation* (*β*)	Standard error (*β*)	95% Confidence interval		*P*
Exhaustion before sleep	0.15	0.15	−0.15	0.44	0.32
Mental balance before sleep	0.00	0.18	−0.35	0.36	0.99
Nocturnal psychosomatic symptoms	0.03	0.09	−0.15	0.21	0.73
Recuperation after sleep	0.31	0.17	−0.01	0.64	0.06
Sleep quality	0.20	0.19	−0.18	0.58	0.31

*Allocation was coded as follows: 1 = control, 2 = exercise.

All models used baseline values of the outcome as well as minimization factors (sex, age, PHQ9 score, and PSQI score) as covariates and allocation as the independent variable. The coefficient for allocation is the difference of the mean change score in the exercise group compared to the control group.

Next, we investigated the effects of exercise on mood. All subscales of the mood questionnaire showed good internal consistency (Cronbach’s alpha > 0.8) except the subscale contemplativeness (Cronbach’s alpha: 0.47 and 0.64 for pre- and post-intervention, respectively). Hence, we did not further analyze the items of the subscale contemplativeness. ANCOVAs of post-intervention mood, adjusted for pre-intervention levels and minimization factors showed that exercise consistently improved mood. Patients in the intervention group reported higher levels of activation (*β* = 0.85, 95% CI: 0.52 to 1.19, *p* < 0.001), elation (*β* = 0.59, 95% CI: 0.36 to 0.82, *p* < 0.001), and calmness (*β* = 0.49, 95% CI: 0.24 to 0.75, *p* < 0.001), as well as less agitation (*β* = −0.48, 95% CI: −0.85 to −0.12, *p* = 0.005), depressiveness (*β* = −0.40, 95% CI: −0.66 to −0.14, *p* = 0.003), fatigue (*β* = −0.91, 95% CI: −1.13 to −0.45, *p* < 0.001), and anger (*β* = −0.26, 95% CI: −0.51 to −0.01, *p* = 0.04).

We did not find evidence for an effect of allocation on adverse outcomes. There were no serious adverse events in either group. We aggregated the questions on adverse outcomes since they showed satisfactory internal consistency at both time points (Cronbach’s alpha: 0.76 and 0.73). The number of reported symptoms immediately after the intervention did not differ between the groups. However, immediately after the intervention there was a trend toward lower symptom severity in the intervention group (median = 0.29) compared to the control group (median = 0.46; *r* = 0.19, *p* = 0.08). Upon awakening on the day after the intervention, there was no evidence to suggest that the group differed in terms of adverse outcome frequency or intensity.

## Discussion

The main goal of our trial was to investigate the effects of 30 min of moderate aerobic exercise in patients with depression on the subsequent night’s sleep efficiency measured by polysomnography. Secondary goals were to assess the effects on other objectively and subjectively measured sleep outcomes, mood, and adverse effects.

We did not find evidence to suggest that a single bout of moderate aerobic exercise improves polysomnographically or subjectively measured sleep outcomes. The absence of evidence for an effect of allocation was very consistent. None of the sensitivity analyses of the primary outcome nor of the secondary polysomnographic or of the subjective sleep outcomes found a significant effect.

This is the first trial to study the *acute* effects of a single bout of aerobic exercise on sleep in patients with depression to the best of our knowledge. Trials investigating the effects of a single bout of exercise on objectively and subjectively measured sleep in patients with insomnia, however, have found equivocal evidence.

While two trials [70, 71] found no effect of exercise on sleep, three trials found some positive effects on objectively measured sleep [67, 72, 73]. The first trial found beneficial effects on sleep onset latency, total sleep time, and sleep efficiency [67]. The second study observed positive effects on actigraphically measured sleep latency and sleep efficiency [73]. The third investigation showed a reduction in stage shifts during the entire night as well as a reduction in stage shifts, arousal index, and wake stages during the second half of the night following exercise performed in the morning [72]. The different findings cannot be readily explained by moderating factors like exercise intensity, duration, or timing. The only trial in which exercise was implemented in the morning revealed a significant effect [72]. Exercising in the afternoon or evening produced mixed results, with some trials finding beneficial [67, 73] and others finding no effect [70–72].

While two trials [70, 71] found no effect of exercise on sleep, three trials found some positive effects on objectively measured sleep [67, 72, 73]. Passos et al. found beneficial effects on sleep onset latency, total sleep time, and sleep efficiency [67]. Li-Jung et al. observed positive effects on actigraphically measured sleep latency and sleep efficiency [73]. Morita et al. showed a reduction in stage shifts during the entire night as well as a reduction in stage shifts, arousal index, and wake stages during the second half of the night following exercise performed in the morning [72]. The different findings cannot be readily explained by moderating factors such as intensity, duration, or timing of the exercise. The only trial in which exercise was implemented during the morning hours revealed a significant effect [72]. Exercising in the afternoon or evening produced mixed results, with some trials finding beneficial [67, 73] and others finding no effect [70–72].

There are several explanations as to why we did not find evidence for the effect of exercise on sleep in this trial. First, issues of internal consistency might have played a role. However, our study design makes this explanation unlikely for the following reasons. We can rule out contamination from other physical activity since step count did not differ between groups and step count was not a significant covariate. There were no differences between the groups at baseline. Moreover, the in-patient rehabilitation setting limits the variability of behavioral aspects which can influence sleep. Second, the trial might have been underpowered due to inappropriate assumptions. Our sample size calculation is based on an effect size for aerobic exercise found in patients with insomnia (a detailed rationale of the sample size calculation can be found in the protocol [25]). However, in our study, polysomnographic outcomes were within the range of healthy individuals [74, 75]. This finding is most likely due to the exclusion of patients who regularly used hypnotics, thus excluding patients with severe insomnia. Reported effect sizes of aerobic exercise on sleep in healthy individuals are smaller [76] than the effect size we used for our sample size calculation. Hence, it is possible that our trial was underpowered to detect a significant difference. Third, a single bout of aerobic exercise might not affect sleep in patients with depression. We cannot provide evidence for the absence of an effect in superiority trials. Thus a non-inferiority trial is needed to provide evidence for the second or third explanation.

The immediate effects of the exercise intervention on mood states were consistently positive. Negative mood states decreased, notably including depressiveness, and positive mood states increased. Findings on the acute effects of moderate aerobic exercise on mood states in patients with depression have been equivocal to a certain degree. While all trials have found positive effects for at least some mood subscales, some have found positive effects on all mood subscales. Our findings are consistent with Niedermeier et al. [77], which also found increased positive and decreased negative mood states with large effect sizes. Stark et al. [78], Frühauf et al. [79], Bartholomew et al. [80], and Legrand et al. [81] also found positive effects on some but not all mood states. Of note, the trial of Stark et al. [78] implemented the same questionnaire as we did, but the intervention lasted 60 min. They found significant and substantial beneficial effects for all subscales except anger. The inconsistencies between the studies mentioned above are likely due to small sample sizes and different outcome questionnaires, which measure slightly different facets of mood.

Adverse outcome severity immediately after the intervention tended to be slightly lower in the intervention group. Although this was a nonsignificant trend and the effect size was small, it is important to note that there is no evidence that exercise increased pain, dizziness, nausea, or cardiovascular and respiratory symptoms. Adverse events are a central aspect of clinical decision-making [46]. However, adverse effects (i.e. an undesirable symptom or outcome temporally associated with an intervention) are underreported in exercise [82] and sleep [46] trials. There are no trials on the acute effects of exercise, which included patients with depression and reported adverse outcomes to the authors’ knowledge. The meta-analysis of Krogh et al. [83] analyzed the chronic effects of exercise in patients with depression. Only approximately 10% and 30% of the included trials reported data on serious and nonserious adverse events, respectively. Based on this limited data, Krogh et al. found that allocation to exercise interventions was associated with a lower risk of nonserious but an increased risk of serious adverse events [83]. The meta-analysis of Niemeijer et al. found no evidence of an increased risk for nonserious adverse events in the psychiatric subgroup [82]. Thus, our study helps to close the gap in the literature concerning the adverse effects of exercise.

This study has several strengths. We took several measures to minimize the risk of bias. These include using minimization (a restricted randomization technique) as well as solid allocation concealment (selection bias), blinding scorers of polysomnographic data (detection bias), quantifying contamination through other physical activity (performance bias), and intent-to-treat analysis (attrition bias). We also carefully selected secondary outcomes which help to inform clinicians, patients, and policymakers. Both scorers have demonstrated good agreement with gold standard ratings of the AASM inter-scorer program [52]. The inter-rater agreement in this trial was within the range reported by other sleep centers [84, 85]. Furthermore, the absence of evidence for an effect of allocation on sleep outcomes and the strong positive effect on the different mood subscales are very consistent.

Limitations include the restricted external validity and the limited polysomnographic montage. The inclusion of patients with psychiatric and somatic comorbidities enhanced the external validity of this study. However, we excluded many of the screened patients because of hypnotics. Although this increased internal validity, it limited external validity. The limits to external validity should be considered when using these findings to inform clinical practice. It is unclear whether the present findings are transferable to patients who regularly use hypnotics. We also made a conscious trade-off between feasibility (reduced polysomnographic montage) and the resulting loss of information. The reduced montage did not allow us to analyze EEG microarchitecture. The strengths and limitations highlighted above point to interesting avenues for future research.

The findings of our study have several scientific implications worth mentioning. Future trials can improve our understanding of exercises’ effects on sleep in patients with depression in many ways. Effectiveness trials could compare the acute effects of different interventions commonly used in in-patient or outpatient treatment settings (e.g. relaxation or mindfulness interventions, light therapy). Importantly, these trials should include patients who use hypnotics. A particularly promising line of investigation is whether exercise is an effective add-on treatment to psychotherapy, pharmacotherapy, or both. The effect of exercise timing is also interesting. The timing of exercise throughout the day seems to alter exercises’ effects on sleep in healthy individuals [76]. This finding might be partially explained by the different effects morning and evening exercise have on melatonin secretion at 10:00 pm [86] and on circadian phase shifts (the latter also depends on the chronotypes) [87]. Any trial on the effects of exercise in patients with depression should systematically collect and report data on adverse effects. Non-inferiority trials could show that exercise does not increases the risk of adverse outcomes. Despite the remaining research questions, this trial can improve clinical decision-making.

These findings can inform clinical practice in multiple ways. A single bout of moderate aerobic exercise will improve mood states and is likely not to have harmful effects on sleep or other symptoms. In addition, patients can expect positive effects on sleep (and many other outcomes, including depressiveness) if they continue to exercise over multiple weeks or months. Our findings also add to the growing body of evidence that exercise performed after 02:00 pm does not reduce sleep quality. This body of evidence is in contrast to current sleep hygiene recommendations [21]. Meta-analyses in healthy populations of all ages have also consistently confirmed that exercise after 02:00 pm either has no effect or even improves sleep [23, 76, 88]. Trials focusing on patients with insomnia have found similar results, although there are far fewer trials available [67, 70–73]. This is relevant to therapeutic settings where exercise interventions are commonly also implemented after 02:00 pm.

## Conclusions

In conclusion, this trial suggests that a single bout of moderate aerobic exercise strongly improves mood but found no evidence for an effect on the subsequent night’s sleep or adverse outcomes. This is the first trial to study the effects of a single bout of exercise on sleep in patients with depression to the best of our knowledge. Additional non-inferiority trials are needed to confirm that moderate aerobic exercise does not negatively affect sleep nor increase the risk of adverse outcomes.

## Supplementary Material

zsab177_suppl_Supplementary_Materials

## References

[1] Edinger JD , et al; American Academy of Sleep Medicine Work Group. Derivation of research diagnostic criteria for insomnia: report of an American Academy of Sleep Medicine Work Group. Sleep. 2004;27(8):1567–1596. doi:10.1093/sleep/27.8.1567. 15683149

[2] Li L , et al Insomnia and the risk of depression: a meta-analysis of prospective cohort studies. BMC Psychiatry. 2016;16(1):375. 27816065 10.1186/s12888-016-1075-3 PMC5097837

[3] Blanken TF , et al Network outcome analysis identifies difficulty initiating sleep as a primary target for prevention of depression: a 6-year prospective study. Sleep. 2020;43(5). doi:10.1093/sleep/zsz288. PMC7215262 31789381

[4] Spiegelhalder K , et al Comorbid sleep disorders in neuropsychiatric disorders across the life cycle. Curr Psychiatry Rep. 2013;15(6):364. 23636987 10.1007/s11920-013-0364-5

[5] Alvaro PK , et al A systematic review assessing bidirectionality between sleep disturbances, anxiety, and depression. Sleep. 2013;36(7):1059–1068. doi:10.5665/sleep.2810. 23814343 PMC3669059

[6] Franzen PL , et al Sleep disturbances and depression: risk relationships for subsequent depression and therapeutic implications. Dialogues Clin Neurosci. 2008;10(4):473–481. 19170404 10.31887/DCNS.2008.10.4/plfranzen PMC3108260

[7] Boland EM , et al Is sleep disturbance linked to short- and long-term outcomes following treatments for recurrent depression? J Affect Disord. 2020;262:323–332. 31735410 10.1016/j.jad.2019.10.033 PMC6919563

[8] Troxel WM , et al Insomnia and objectively measured sleep disturbances predict treatment outcome in depressed patients treated with psychotherapy or psychotherapy-pharmacotherapy combinations. J Clin Psychiatry. 2012;73(4):478–485. 22152403 10.4088/JCP.11m07184 PMC3310298

[9] Mason BL , et al Focusing on insomnia symptoms to better understand depression: a STAR*D report. J Affect Disord. 2020;260:183–186. 31499373 10.1016/j.jad.2019.08.094 PMC6803100

[10] Cepeda MS , et al Finding factors that predict treatment-resistant depression: results of a cohort study. Depress Anxiety. 2018;35(7):668–673. 29786922 10.1002/da.22774 PMC6055726

[11] Wang X , et al Systematic review and meta-analysis of the relationship between sleep disorders and suicidal behaviour in patients with depression. BMC Psychiatry. 2019;19(1):303. 31623600 10.1186/s12888-019-2302-5 PMC6798511

[12] Carney RM , et al Insomnia and depression prior to myocardial infarction. Psychosom Med. 1990;52(6):603–609. 2287700 10.1097/00006842-199011000-00001

[13] van Mill JG , et al Insomnia and sleep duration in a large cohort of patients with major depressive disorder and anxiety disorders. J Clin Psychiatry. 2010;71(3):239–246. 20331928 10.4088/JCP.09m05218gry

[14] Perlis ML , et al Self-reported sleep disturbance as a prodromal symptom in recurrent depression. J Affect Disord. 1997;42(2-3):209–212. 9105962 10.1016/s0165-0327(96)01411-5

[15] Nierenberg AA , et al Residual symptoms after remission of major depressive disorder with citalopram and risk of relapse: a STAR*D report. Psychol Med. 2010;40(1):41–50. 19460188 10.1017/S0033291709006011 PMC5886713

[16] Norton K , et al Position statement on physical activity and exercise intensity terminology. J Sci Med Sport. 2010;13(5):496–502. 20005170 10.1016/j.jsams.2009.09.008

[17] Brupbacher G , et al The effects of exercise on sleep in unipolar depression: a systematic review and network meta-analysis. Sleep Med Rev. 2021;59:101452. 33667885 10.1016/j.smrv.2021.101452

[18] Morres ID , et al Aerobic exercise for adult patients with major depressive disorder in mental health services: a systematic review and meta-analysis. Depress Anxiety. 2019;36(1):39–53. 30334597 10.1002/da.22842

[19] Stubbs B , et al Exercise improves cardiorespiratory fitness in people with depression: a meta-analysis of randomized control trials. J Affect Disord. 2016;190:249–253. 26523669 10.1016/j.jad.2015.10.010

[20] Gan Y , et al Depression and the risk of coronary heart disease: a meta-analysis of prospective cohort studies. BMC Psychiatry. 2014;14:371. 25540022 10.1186/s12888-014-0371-z PMC4336481

[21] American Sleep Association. Sleep Hygiene Tips. Boston, MA: American Sleep Association. https://www.sleepassociation.org/about-sleep/sleep-hygiene-tips/. Accessed May 18, 2021.

[22] Buman MP , et al Does nighttime exercise really disturb sleep? Results from the 2013 National Sleep Foundation Sleep in America Poll. Sleep Med. 2014;15(7):755–761. 24933083 10.1016/j.sleep.2014.01.008

[23] Stutz J , et al Effects of evening exercise on sleep in healthy participants: a systematic review and meta-analysis. Sports Med. 2019;49(2):269–287. 30374942 10.1007/s40279-018-1015-0

[24] Haskell WL , et al; American College of Sports Medicine; American Heart Association. Physical activity and public health: updated recommendation for adults from the American College of Sports Medicine and the American Heart Association. Circulation. 2007;116(9):1081–1093. 17671237 10.1161/CIRCULATIONAHA.107.185649

[25] Brupbacher G , et al The acute effects of aerobic exercise on sleep in patients with depression: study protocol for a randomized controlled trial. Trials. 2019;20(1):352. 31196147 10.1186/s13063-019-3415-3 PMC6567535

[26] Schulz KF , et al; CONSORT Group. CONSORT 2010 Statement: updated guidelines for reporting parallel group randomised trials. BMC Med. 2010;8:18. 20334633 10.1186/1741-7015-8-18 PMC2860339

[27] Dickhuth HH , et al Ventilatory, lactate-derived and catecholamine thresholds during incremental treadmill running: relationship and reproducibility. Int J Sports Med. 1999;20(2):122–127. 10190774 10.1055/s-2007-971105

[28] Kroenke K , et al The PHQ-15: validity of a new measure for evaluating the severity of somatic symptoms. Psychosom Med. 2002;64(2):258–266. 11914441 10.1097/00006842-200203000-00008

[29] Linn BS , et al Cumulative illness rating scale. J Am Geriatr Soc. 1968;16(5):622–626. 5646906 10.1111/j.1532-5415.1968.tb02103.x

[30] Obbarius A , et al Standardization of health outcomes assessment for depression and anxiety: recommendations from the ICHOM Depression and Anxiety Working Group. Qual Life Res. 2017;26(12):3211–3225. 28786017 10.1007/s11136-017-1659-5 PMC5681977

[31] Herrmann C , et al HADS-D – Hospital Anxiety and Depression Scale – Deutsche Version (HADS-D – Hospital Anxiety and Depression Scale – German Version). Bern, Switzerland: Huber; 2011.

[32] Brennan C , et al The Hospital Anxiety and Depression Scale: a diagnostic meta-analysis of case-finding ability. J Psychosom Res. 2010;69(4):371–378. 20846538 10.1016/j.jpsychores.2010.04.006

[33] Klein EM , et al The German version of the Perceived Stress Scale – psychometric characteristics in a representative German community sample. BMC Psychiatry. 2016;16:159. 27216151 10.1186/s12888-016-0875-9 PMC4877813

[34] Drake C , et al Vulnerability to stress-related sleep disturbance and hyperarousal. Sleep. 2004;27(2):285–291. doi:10.1093/sleep/27.2.28. 15124724

[35] Kalmbach DA , et al Identifying at-risk individuals for insomnia using the Ford Insomnia Response to Stress Test. Sleep. 2016;39(2):449–456. doi:10.5665/sleep.5462. 26446111 PMC4712406

[36] Dieck A , et al Validation of the German version of the Ford Insomnia Response to Stress Test. J Sleep Res. 2018;27(3):e12621. 29047222 10.1111/jsr.12621

[37] Lang C , et al Validation of the German version of the short form of the dysfunctional beliefs and attitudes about sleep scale (DBAS-16). Neurol Sci. 2017;38(6):1047–1058. doi:10.1007/s10072-017-2921-x. 28321516

[38] Horne JA , et al A self-assessment questionnaire to determine morningness-eveningness in human circadian rhythms. Int J Chronobiol. 1976;4(2):97–110. 1027738

[39] Kantermann T , et al Comparing the Morningness-Eveningness Questionnaire and Munich ChronoType Questionnaire to the dim light melatonin onset. J Biol Rhythms. 2015;30(5):449–453. 26243627 10.1177/0748730415597520 PMC4580371

[40] Griefahn B , et al Zur Validität der deutschen Übersetzung des Morningness-Eveningness-Questionnaires von Horne und Östberg. Somnologie. 2001;5(2):71–80. doi:10.1046/j.1439-054X.2001.01149.x.

[41] Johns MW . A new method for measuring daytime sleepiness: the Epworth sleepiness scale. Sleep. 1991;14(6):540–545. doi:10.1093/sleep/14.6.540. 1798888

[42] Bloch KE , et al German version of the Epworth Sleepiness Scale. Respiration. 1999;66(5):440–447. 10516541 10.1159/000029408

[43] Buysse DJ , et al The Pittsburgh Sleep Quality Index: a new instrument for psychiatric practice and research. Psychiatry Res. 1989;28(2):193–213. 2748771 10.1016/0165-1781(89)90047-4

[44] Backhaus J , et al Schlafstörungen Bewältigen [Coping with Sleep Disorders]. Weinheim, Germany: Beltz Psychologie Verlags Union; 1996. https://scholar.google.com/scholar_lookup?title=Schlafst%C3%B6rungen%20bew%C3%A4ltigen%20%5BCoping%20with%20sleep%20disorders%5D&author=J.%20Backhaus&author=D.%20Riemann&publication_year=1996. Accessed February 20, 2018.

[45] Chiu HY , et al A meta-analysis of diagnostic accuracy of three screening tools for insomnia. J Psychosom Res. 2016;87:85–92. 27411756 10.1016/j.jpsychores.2016.06.010

[46] Sateia MJ , et al Clinical practice guideline for the pharmacologic treatment of chronic insomnia in adults: an American Academy of Sleep Medicine Clinical Practice Guideline. J Clin Sleep Med. 2017;13(2):307–349. 27998379 10.5664/jcsm.6470 PMC5263087

[47] Morgenthaler T , et al; American Academy of Sleep Medicine. Practice parameters for the psychological and behavioral treatment of insomnia: an update. An American Academy of Sleep Medicine report. Sleep. 2006;29(11):1415–1419. doi:10.1093/sleep/29.11.1415. 17162987

[48] Devilly GJ , et al Psychometric properties of the credibility/expectancy questionnaire. J Behav Ther Exp Psychiatry. 2000;31(2):73–86. 11132119 10.1016/s0005-7916(00)00012-4

[49] Williams AD , et al Combining imagination and reason in the treatment of depression: a randomized controlled trial of internet-based cognitive-bias modification and internet-CBT for depression. J Consult Clin Psychol. 2013;81(5):793–799. 23750459 10.1037/a0033247 PMC3780629

[50] Fietze I , et al Actigraphy combined with EEG compared to polysomnography in sleep apnea patients. Physiol Meas. 2015;36(3):385–396. 25651914 10.1088/0967-3334/36/3/385

[51] Berry RB , et al The AASM Manual for the Scoring of Sleep and Associated Events: Rules, Terminology and Technical Specifications. Darien, IL: American Academy of Sleep Medicine; 2012. http://www.aasmnet.org/resources/pdf/scoring-manual-preface.pdf. Accessed May 5, 2017.

[52] Kendrick AH , et al Quality assurance of full polysomnography scoring using the American Academy of Sleep Medicine (AASM) inter-scorer reliability (ISR) program. Eur Respir J. 2017;50(Suppl 61):PA2485. doi:10.1183/1393003.congress-2017.PA2485.

[53] Rosenberg RS , et al The American Academy of Sleep Medicine inter-scorer reliability program: sleep stage scoring. J Clin Sleep Med. 2013;9(1):81–87. 23319910 10.5664/jcsm.2350 PMC3525994

[54] Görtelmeyer R. Schlaffragebogen A Und B: SF-A/R Und SF-B/R. Göttingen, Germany: Hogrefe; 2011.

[55] Riemann D , et al S3-Leitlinie Nicht erholsamer Schlaf/Schlafstörungen. Somnologie. 2017;21(1):2–44.

[56] O’Callaghan CA . OxMaR: open source free software for online minimization and randomization for clinical trials. PLoS One. 2014;9(10):e110761. 25353169 10.1371/journal.pone.0110761 PMC4213009

[57] Chan AW , et al SPIRIT 2013 statement: defining standard protocol items for clinical trials. Ann Intern Med. 2013;158(3):200–207. 23295957 10.7326/0003-4819-158-3-201302050-00583 PMC5114123

[58] Abele-Brehm A , Brehm W. Zur Konzeptualisierung und Messung von Befindlichkeit: Die Entwicklung der “Befindlichkeitsskalen” (BFS) [The conceptualization and measurement of mood: the development of the “Mood Survey.”]. Diagnostica. 1986;32(3):209–228.

[59] Höchsmann C , et al Validity of activity trackers, smartphones, and phone applications to measure steps in various walking conditions. Scand J Med Sci Sports. 2018;28(7):1818–1827. 29460319 10.1111/sms.13074

[60] MacLean AW , et al. Psychometric evaluation of the Stanford Sleepiness Scale. J Sleep Res. 1992;1(1):35–39. 10607023 10.1111/j.1365-2869.1992.tb00006.x

[61] Baglioni C , et al Sleep and mental disorders: a meta-analysis of polysomnographic research. Psychol Bull. 2016;142(9):969–990. 27416139 10.1037/bul0000053 PMC5110386

[62] Kahan BC , et al Reporting and analysis of trials using stratified randomisation in leading medical journals: review and reanalysis. BMJ. 2012;345:e5840. 22983531 10.1136/bmj.e5840 PMC3444136

[63] Long JS , Ervin LH. Using heteroscedasticity consistent standard errors in the linear regression model. Am Stat. 2000;54(3):217–224. doi:10.1080/00031305.2000.10474549

[64] van Buuren S , Groothuis-Oudshoorn K. MICE: multivariate imputation by chained equations in R. J Stat Softw. 2011;45(1):1–67. doi:10.18637/jss.v045.i03

[65] R Core Team. R: A Language and Environment for Statistical Computing. Vienna, Austria: R Foundation for Statistical Computing; 2020. https://www.R-project.org/.

[66] Borm GF , et al A simple sample size formula for analysis of covariance in randomized clinical trials. J Clin Epidemiol. 2007;60(12):1234–1238. 17998077 10.1016/j.jclinepi.2007.02.006

[67] Passos GS , et al Effect of acute physical exercise on patients with chronic primary insomnia. J Clin Sleep Med. 2010;6(3):270–275. 20572421 PMC2883039

[68] Stubbs B , et al Dropout from exercise randomized controlled trials among people with depression: a meta-analysis and meta regression. J Affect Disord. 2016;190:457–466. 26551405 10.1016/j.jad.2015.10.019

[69] Benjamini Y , Hochberg Y. Controlling the false discovery rate: a practical and powerful approach to multiple testing. J R Stat Soc Series B Stat Methodol. 1995;5(1):289–300.

[70] Youngstedt SD , et al Testing the sleep hygiene recommendation against nighttime exercise. Sleep Breath. 2021. doi:10.1007/s11325-020-02284-x. 33423141

[71] Baron KG , et al Exercise to improve sleep in insomnia: exploration of the bidirectional effects. J Clin Sleep Med. 2013;9(8):819–824. 23946713 10.5664/jcsm.2930 PMC3716674

[72] Morita Y , et al Effects of acute morning and evening exercise on subjective and objective sleep quality in older individuals with insomnia. Sleep Med. 2017;34:200–208. 28522092 10.1016/j.sleep.2017.03.014

[73] Chen LJ , et al Effects of an acute bout of light-intensity walking on sleep in older women with sleep impairment: a randomized controlled trial. J Clin Sleep Med. 2019;15(4):581–586. 30952217 10.5664/jcsm.7718 PMC6457511

[74] Hertenstein E , et al Reference data for polysomnography-measured and subjective sleep in healthy adults. J Clin Sleep Med. 14(04):523–532. doi:10.5664/jcsm.7036 29609718 PMC5886429

[75] Boulos MI , et al Normal polysomnography parameters in healthy adults: a systematic review and meta-analysis. Lancet Respir Med. 2019;7(6):533–543. 31006560 10.1016/S2213-2600(19)30057-8

[76] Kredlow MA , et al The effects of physical activity on sleep: a meta-analytic review. J Behav Med. 2015;38(3):427–449. 25596964 10.1007/s10865-015-9617-6

[77] Niedermeier M , et al Acute effects of a single bout of walking on affective responses in patients with major depressive disorder. Int J Environ Res Public Health. 2021;18(4):1524. doi:10.3390/ijerph18041524. 33562699 PMC7914602

[78] Stark R , et al Acute effects of a single bout of moderate exercise on psychological well-being in patients with affective disorder during hospital treatment. Neuropsychiatr. 2012;26(4):166–170. 23111811 10.1007/s40211-012-0033-7

[79] Frühauf A , et al Acute effects of outdoor physical activity on affect and psychological well-being in depressed patients – a preliminary study. Ment Health Phys Act. 2016;10:4–9. doi:10.1016/j.mhpa.2016.02.002.

[80] Bartholomew JB , et al Effects of acute exercise on mood and well-being in patients with major depressive disorder. Med Sci Sports Exerc. 2005;37(12):2032–2037. 16331126 10.1249/01.mss.0000178101.78322.dd

[81] Legrand FD , et al Acute effects of outdoor and indoor exercise on feelings of energy and fatigue in people with depressive symptoms. J Environ Psychol. 2018;56:91–96. doi:10.1016/j.jenvp.2018.03.005.

[82] Niemeijer A , et al Adverse events of exercise therapy in randomised controlled trials: a systematic review and meta-analysis. Br J Sports Med. 2020;54(18):1073–1080. 31563884 10.1136/bjsports-2018-100461

[83] Krogh J , et al Exercise for patients with major depression: a systematic review with meta-analysis and trial sequential analysis. BMJ Open. 2017;7(9):e014820. 10.1136/bmjopen-2016-014820 PMC5623558 28928174

[84] Danker-Hopfe H , et al Interrater reliability for sleep scoring according to the Rechtschaffen & Kales and the new AASM standard. J Sleep Res. 2009;18(1):74–84. 19250176 10.1111/j.1365-2869.2008.00700.x

[85] Zhang X , et al Process and outcome for international reliability in sleep scoring. Sleep Breath. 2015;19(1):191–195. 24801137 10.1007/s11325-014-0990-0

[86] Carlson LA , et al Influence of exercise time of day on salivary melatonin responses. Int J Sports Physiol Perform. 2019;14(3):351–353. 30160559 10.1123/ijspp.2018-0073

[87] Thomas JM , et al Circadian rhythm phase shifts caused by timed exercise vary with chronotype. JCI Insight. 2020;5(3). doi:10.1172/jci.insight.134270. PMC7098792 31895695

[88] Seol J , et al Effects of morning versus evening home-based exercise on subjective and objective sleep parameters in older adults: a randomized controlled trial. J Geriatr Psychiatry Neurol. 2020;34(3):232–242. doi:10.1177/0891988720924709. 32431208

