# Supplementary material for: The acute effects of aerobic exercise on sleep in patients with unipolar depression: a randomized controlled trial
Source: Sleep. 2021 Jul 13;44(11):zsab177. doi: 10.1093/sleep/zsab177 (PMC8598185; doi:10.1093/sleep/zsab177)
Supplement: zsab177_suppl_Supplementary_Materials [file zsab177_suppl_supplementary_materials.docx]

**The acute effects of aerobic exercise on sleep in patients with unipolar depression: a randomized controlled trial**

Authors and author affiliations:

Gavin Brupbacher ^1 2^, Thea Zander-Schellenberg ^3^, Doris Straus ^2^, Hildburg Porschke ^2^, Denis Infanger ^1^, Markus Gerber ^4^, Roland von Känel ^5^, Arno Schmidt-Trucksäss ^1^

^1^Division of Sports and Exercise Medicine, Department of Sport, Exercise and Health, University of Basel, Birsstrasse 320 B, 4052, Basel, Switzerland.

^2^OBERWAID AG, Rorschacher Strasse 311, 9016, St. Gallen, Switzerland.

^3^Department of Psychology, Division of Clinical Psychology and Epidemiology, University of Basel, Basel, Switzerland.

^4^Division of Sport and Psychosocial Health, Department of Sport, Exercise and Health, University of Basel, Birsstrasse 320 B, 4052, Basel, Switzerland.

^5^Department of Consultation-Liaison Psychiatry and Psychosomatic Medicine, University Hospital Zurich, Culmannstrasse 8, 8091, Zurich, Switzerland.

Corresponding author:

Gavin Brupbacher

^1^Division of Sports and Exercise Medicine, Department of Sport, Exercise and Health, University of Basel, Birsstrasse 320 B, 4052, Basel, Switzerland. gavin.brupbacher@unibas.ch.

^2^OBERWAID AG, Rorschacher Strasse 311, 9016, St. Gallen, Switzerland. gavin.brupbacher@oberwaid.ch.

**Supplement**

**Section 1: Minimization scheme of EASED trial**

A nondeterministic unweighted minimization algorithm with a random element of 0.8 will be used to increase the probability of balanced groups. The allocation ratio is 1:1. Allocation to intervention or control group will be done using the open source software for online minimization (Oxford Minimization and Randomization, OxMaR)^1^. The following factors and corresponding classes will be used for minimization:

- sex (male, female)
- age in years (18-26, 27-36, 37-46, 47-56, 57-65)
- depression severity measured by PHQ-9 score (0-4, 5-9, 10-14, 15-19, 20-27)
- sleep quality measured by PSQI score (0-4, 5-10, 11-16, 17-21)

These variables were chosen because previous studies have shown that the effect of exercise on sleep may vary between gender^2^, objectively measured sleep characteristics change linearly throughout adulthood^3^, and sleep quality as well as depression severity might alter effectiveness of the intervention. Age classes are created by dividing the age range as equally as possible in five classes. Depression severity classes are based on validated cut-offs for no, mild, moderate, moderately severe, and severe depression^4^. In the sleep quality factor, the validated cut-off (≥5) is used to delineate the first class^5^. Since most patients are above this cut-off, the remaining range of scores is further divided into even classes.

A simulation using the software SiMin^6^ with 5000 iterations was performed to estimate the discrepancies between the groups using the above mentioned specifications. The mean discrepancies between the exercise and control group are estimated to be 1.83 for gender, 2.59 for PSQI score, and 3.42 for age as well as PHQ-9 score. With a probability of 0.95 the discrepancy between groups will not exceed 4 patients for sex and 5 patients for age, PHQ-9 score, and PSQI-score. The simulation showed that weighting factors caused an increase in the discrepancy of sex while not affecting the other variables. Therefore, factors will not be weighted in the minimization.

**References**

1. O’Callaghan CA. OxMaR: Open Source Free Software for Online Minimization and Randomization for Clinical Trials. *PLOS ONE*. 2014;9(10):e110761. doi:10.1371/journal.pone.0110761

2. Kredlow MA, Capozzoli MC, Hearon BA, Calkins AW, Otto MW. The effects of physical activity on sleep: a meta-analytic review. *J Behav Med*. 2015;38(3):427-449. doi:10.1007/s10865-015-9617-6

3. Ohayon MM, Carskadon MA, Guilleminault C, Vitiello MV. Meta-analysis of quantitative sleep parameters from childhood to old age in healthy individuals: developing normative sleep values across the human lifespan. *Sleep*. 2004;27(7):1255-1273.

4. Kroenke K, Spitzer RL, Williams JB. The PHQ-9: validity of a brief depression severity measure. *J Gen Intern Med*. 2001;16(9):606-613.

5. Buysse DJ, Reynolds CF, Monk TH, Berman SR, Kupfer DJ. The Pittsburgh Sleep Quality Index: a new instrument for psychiatric practice and research. *Psychiatry Res*. 1989;28(2):193-213.

6. Wade A, Pan H, Eaton S, Pierro A, Ong E. An investigation of minimisation criteria. *BMC Med Res Methodol*. 2006;6:11. doi:10.1186/1471-2288-6-11

**Figure S1: Rate of perceived exertion during the intervention**

**
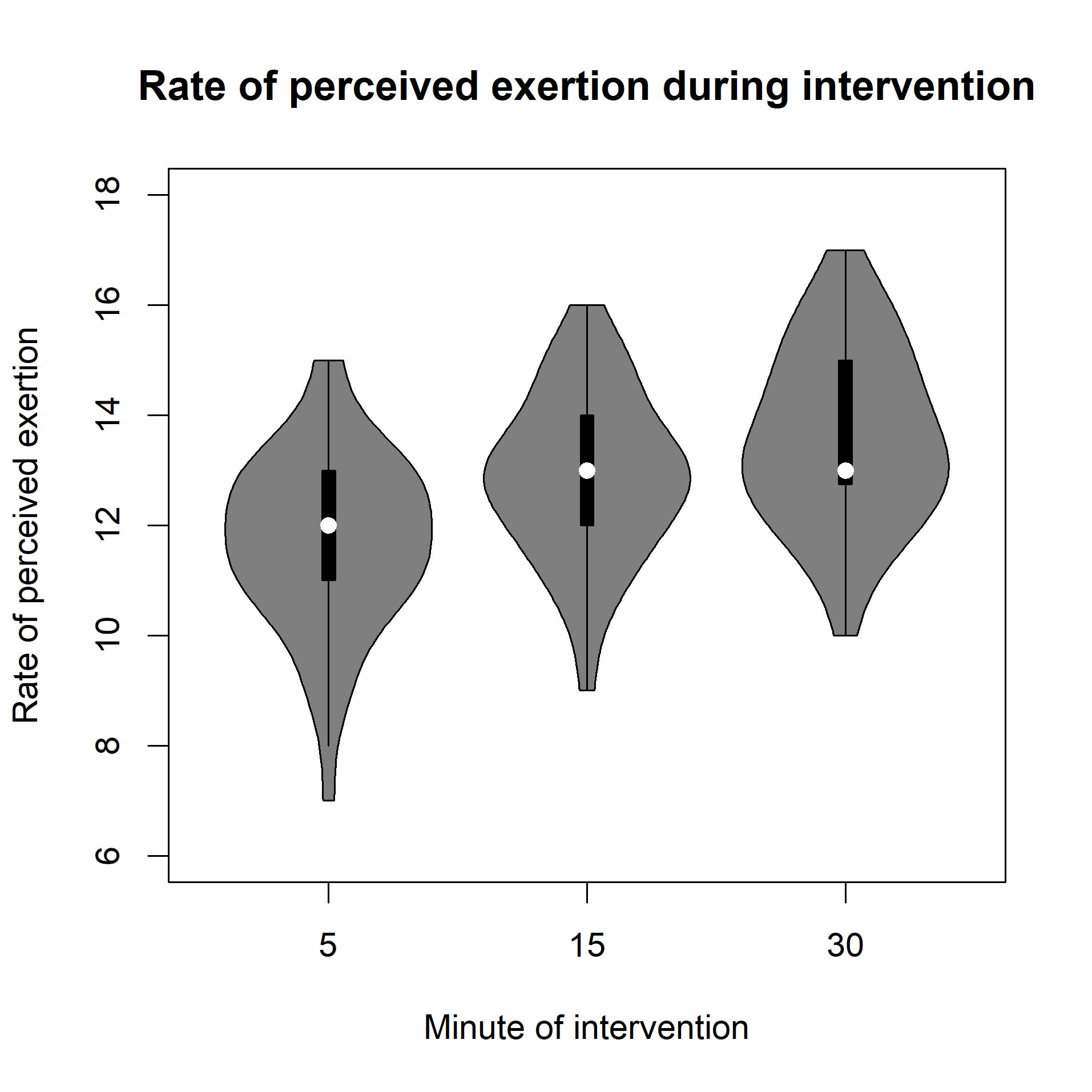
**

Note: rate of perceived exertion is rated on a scale from 6 to 20.

**Figure S2: Average heart rate during the intervention**

**
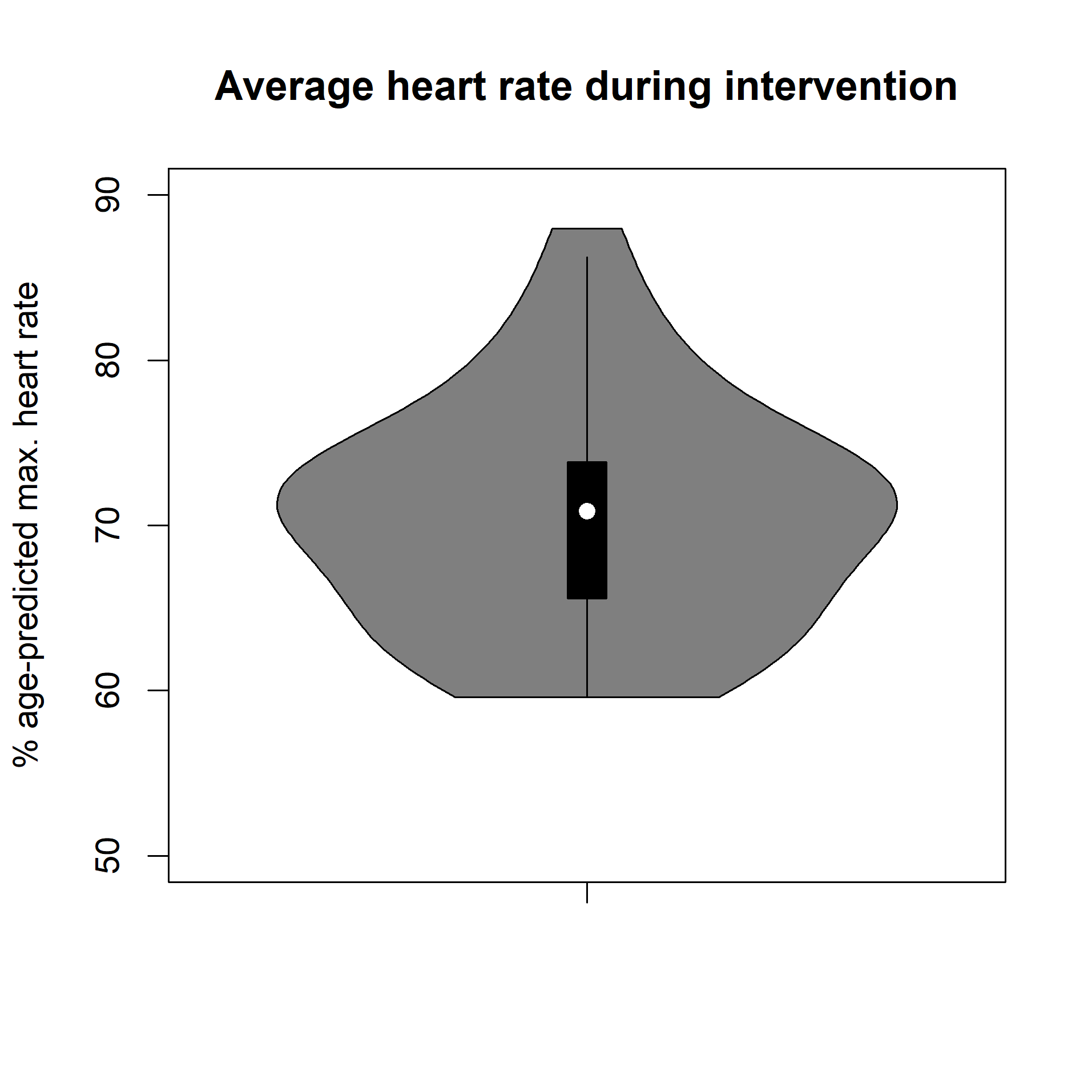
**

Note: age-predicted heart rate is calculated according to the formula of Tanaka et al., 2001, i.e., 208 − 0.7 × age.

**Figure S3: Daytime sleepiness**

**
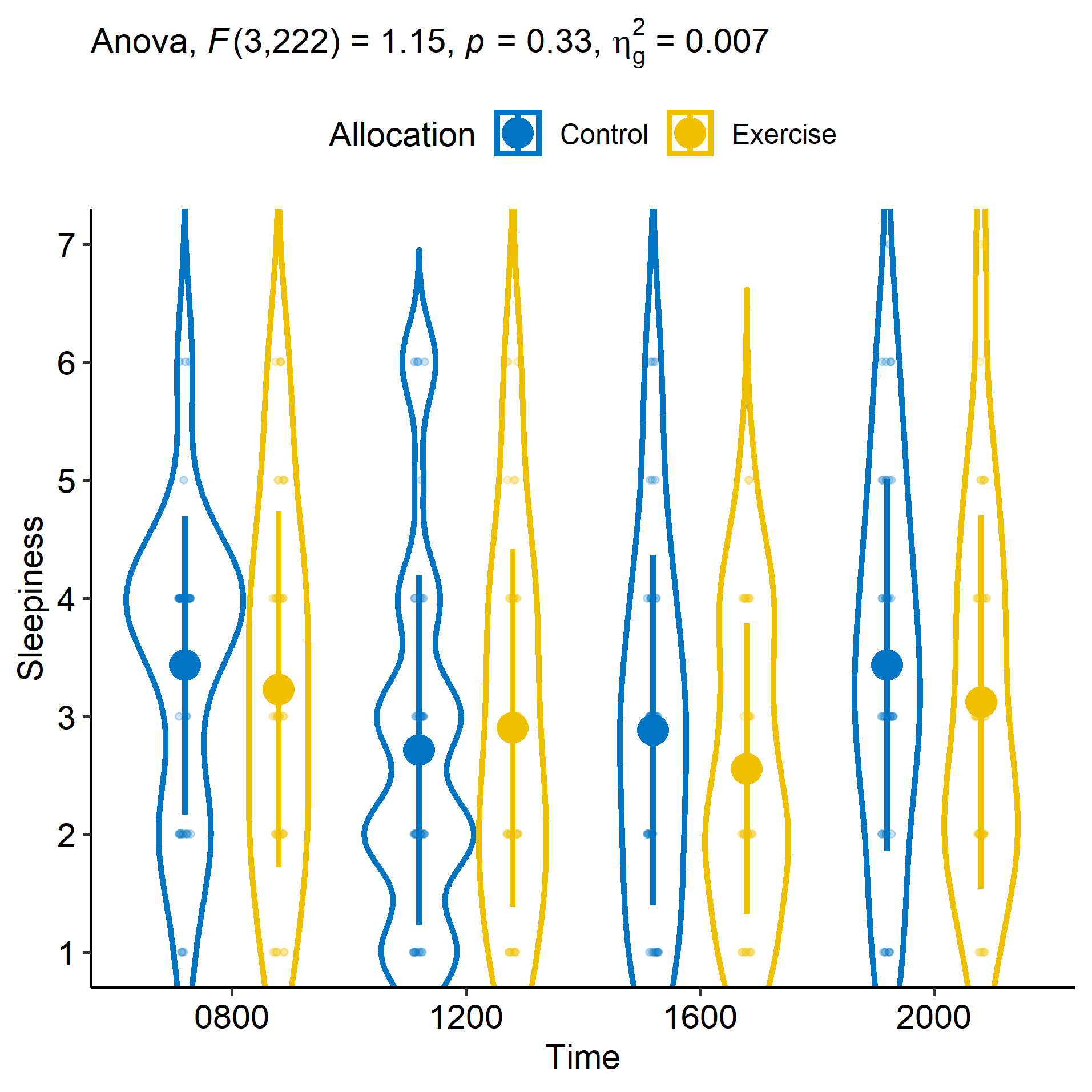
**
